# Reducing biting rates of *Aedes aegypti* with metofluthrin: investigations in time and space

**DOI:** 10.1186/s13071-017-2004-0

**Published:** 2017-02-07

**Authors:** Jonathan M. Darbro, M. Odwell Muzari, Arthur Giblin, Rebecca M. Adamczyk, Scott A. Ritchie, Gregor J. Devine

**Affiliations:** 1 QIMR Berghofer Medical Research Institute, Herston, QLD Australia; 2 Tropical Public Health Services, Cairns, QLD Australia; 3 James Cook University, Cairns, QLD Australia

**Keywords:** Metofluthrin, Volatile pyrethroids, *Aedes aegypti*, Dengue, Vector control

## Abstract

**Background:**

Indoor residual spraying is key to dengue control in Cairns and other parts of northern Queensland, Australia, where *Aedes aegypti* is prevalent, but the strategy faces challenges with regards to slow application time and, therefore, community coverage. A faster potential improvement might be the use of polyethylene netting impregnated with the volatile pyrethroid metofluthrin (SumiOne™). This formulation was assessed in rooms in three houses in Cairns, Australia. One emanator was placed in each room and cages of 10 female *Aedes aegypti* were exposed at distances of 1 and 3 m. Knockdown and landings on a human hand were counted before metofluthrin exposure and at 10, 30, 60, 90 and 120 min during exposure. In addition, two trials continued over 48 h of exposure to assess the long-term sublethal effects of metofluthrin on caged mosquitoes.

**Results:**

Percentage landing rates fell to 0–2.5% in the first 10 min of exposure. Knockdown was most evident between 10 and 30 min (54% at 1 m and 33% at 3 m). Distance from the emanator strongly affected the results: mosquitoes at 3 m exhibited less knockdown and more landings than those at 1 m. As room volume increased, knockdown decreased and the number of landing increased. There is a cumulative mortality and landing inhibition and, for mosquitoes exposed to metofluthrin for > 48 h, mortality was 100% at 1 m and 90% at 3 m. Of those still alive, a small number continued to land and bite. After being removed from metofluthrin-treated rooms, exposed insect cages were found to reducing landing rates for up to 2 h.

**Conclusions:**

Despite only moderate levels of knockdown during the initial hours of exposure, metofluthrin emanators were effective in reducing mosquito landing rates, especially within 1 m, even when exposed on an open veranda. The evaluation methods and results described in this paper will help inform the optimal conditions of deployment of metofluthrin emanators. These devices have the potential to reduce contact between humans and urban disease vectors faster than indoor residual spraying so supplement our current arsenal of dengue control tools.

## Background

Dengue continues to be a public health concern in northern Queensland and much of the tropical world [1]. Outbreaks in northern Queensland, Australia begin with the importation of a viraemic patient infected overseas and are then locally transmitted by endemic populations of *Aedes aegypti*. Given the increase in global traffic and the subsequent number of viraemic imports [2], it is likely that seasonal dengue incidence will continue and may increase. The presence of *Ae. aegypti* also makes parts of north Queensland prone to outbreaks of Zika virus introduced through travellers in the same way as dengue.

Although public health authorities in northern Queensland have been successful in limiting dengue outbreaks in the past, a reliance on labour-intensive indoor residual spraying (IRS) as the primary method of controlling *Ae. aegypti* [3, 4] has remained a challenge to achieving optimal coverage. Limitations with IRS include the time required (and therefore the cost) to treat individual houses. An approximate 20 min treatment time severely limits the number of houses that can be treated by a vector control team in any single day, even without the additional complication of obtaining home owners’ consents for the process. Finally, in many parts of the world, *Ae. aegypti* is resistant to conventional residual pyrethroids [5–7], although there is no evidence of that in Australian populations.

Volatile pyrethroids such as metofluthrin [8], disseminated from a number of point sources and surfaces, at a variety of concentrations, have demonstrated their potential to repel or reduce populations of *Culex* [9–12], *Anopheles* [10, 11, 13, 14] and *Aedes* [9, 15–17]. These kinds of devices (or “emanators”), pre-treated with metofluthrin, might be hung in a room in a fraction of the time it takes to conduct IRS. The speed of deployment, the relative lack of disruption and the absence of long-lasting residues on household surfaces may make homeowners less likely to object to “emanators” than IRS. Previous research in simulated domestic settings in Queensland has shown that emanator use may result in moderate to high lethality of free-flying *Ae. aegypti*, and that those mosquitoes that survive are much less likely to bite [16, 17].

Although there have been studies evaluating metofluthrin in “modified” residential spaces [16, 17], there have been no studies to our knowledge that evaluate these devices in “real world” urban spaces. Here we tested metofluthrin emanators against *Ae. aegypti* in 9 different rooms in 3 residences in northern Queensland to evaluate their efficacy under conditions of operational relevance. We tested: (i) the reduction of mosquito biting activity in the presence of metofluthrin over 2 h; (ii) the knockdown of mosquitoes in the presence of metofluthrin over 2 h; and (iii) biting behaviour of mosquitoes exposed to metofluthrin over an extended period (> 48 h).

## Methods

### Trial houses

Trials took place at three houses in Cairns, Queensland. Three to four rooms were chosen in each house to represent a range of qualities such as volume, ventilation and exit sizes (Table 1). Ceiling fans were kept on their lowest setting in each room to facilitate air movement and the dissemination of metofluthrin around the room. Doors were kept closed whenever possible. Trials were carried out between November 2013 and November 2016.

**Table 1:** Types and volumes of rooms used in field trials

House	Room	Dimensions (m)	Volume (m^3^)
1	Lounge	4.5 × 4.5 × 2.5	50.6
	Bedroom 1	3.5 × 3.0 × 2.5	26.3
	Bedroom 2	3.5 × 3.0 × 2.5	26.3
	Veranda	Not measured	> 100
2	Bedroom 1	3.8 × 3.5 × 3.3	43.9
	Bedroom 2	4.0 × 4.9 × 2.1	41.2
	Bedroom 3	3.5 × 5.4 × 2.0	37.8
	Lounge	3.8 × 3.5 × 3.3	43.9
	Veranda	> 7 × 3 × > 3	> 63
	Carport	Not measured	> 100
3	Lounge	11.9 × 3.8 × 2.7	122.1
	Main room	4.2 × 3.8 × 2.7	43.1
	Spare room	3.9 × 3.8 × 2.7	40.0
	Area under house	Not measured	> 100

House #1 included the following rooms: lounge (4.5 × 4.5 × 2.5 m), two bedrooms (each 3.5 × 3.0 × 2.5 m) and a veranda (> 100 m^3^). Temperature ranged between 23 °C and 33 °C.

House #2 trials took place in either a guest bedroom (3.8 × 3.5 × 3.3 m), the TV lounge (3.8 × 3.5 × 3.3 m), the veranda (> 7 × 3 × > 3.0 m) or the carport (> 100 m^3^). Temperature ranged between 21 °C and 31 °C.

Efficacy trials in House #3 and residual persistence trials took place a lounge (11.9 × 3.8 × 2.7 m), a utility room (4.2 × 3.8 × 2.7 m), a spare bedroom (3.9 × 3.8 × 2.7 m) and the space under the house (> 100 m^3^). Temperature ranged between 22 °C and 31 °C.

### Mosquitoes

*Aedes aegypti* mosquitoes were from a colony sourced from ovitraps around Cairns, and the mosquitoes had been in colony at James Cook University, Cairns, for three generations. Although mosquitoes infected with the endosymbiont *Wolbachia* have been released in several suburbs in Cairns [18], the mosquitoes in this study were collected from suburbs where *Wolbachia* had not been released.

### Metofluthrin

**Fig. 1:**
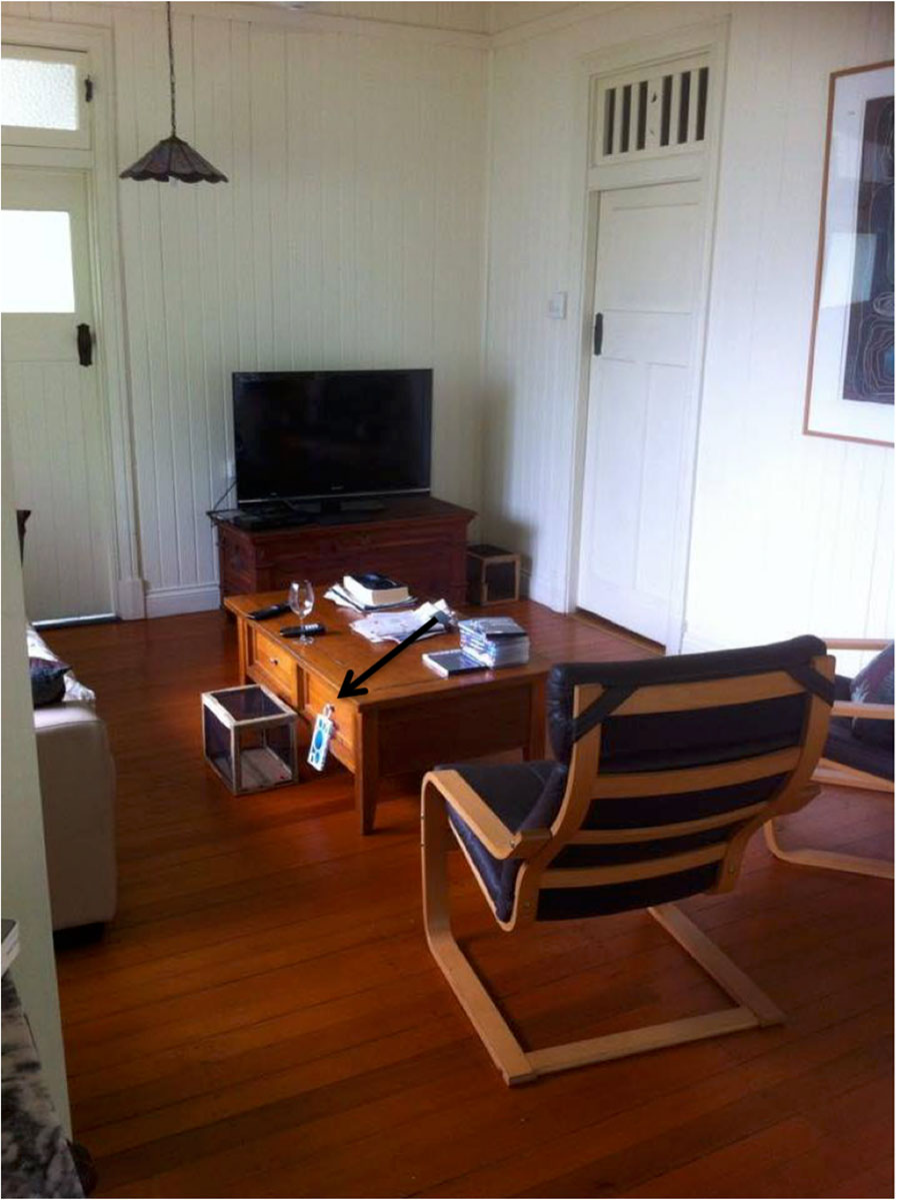
One of the trial rooms. Lounge, House #2. One mosquito cage is within 1 m of the metofluthrin emanator (*arrow*) and the other cage is 3 m away

Devices consisting of polyethylene netting impregnated with the volatile pyrethroid metofluthrin (SumiOne™) were provided by Sumitomo Chemical Australia Pty Limited. The netting contained 10% metofluthrin by weight and a previous study has shown that they maintain full activity for at least 20 days (Ritchie & Devine 2013). This formulation was approved by the Australian Pesticides and Veterinary Medicines Authority in 2015 (reference 70086/62469). We re-used emanators for ≤ 14 days during this study. In each room used for the evaluations, emanators were hung from or under furniture approximately 10–30 cm off the ground (Fig. 1).

### Efficacy trials

Ten adult, female *Aedes aegypti* (4–7 days old) were placed in 30 cm^3^ cardboard and polypropylene mesh cages (9 × 9 mesh/m^2^). They were given 15 min to acclimate, in the absence of metofluthrin exposure, before a preliminary assessment of knockdown and landing rate. A cage of mosquitoes was only used if > 80% of the mosquitoes in these pre-exposure assays attempted to feed within 2 min. Knockdown was evaluated by counting the number of mosquitoes that were not able to fly when the cage was lightly disturbed. After the pre-exposure counts, the cages were moved to rooms within each house. In each room, one cage was placed < 1 m from the emanator, and the other was placed 3 m away. All cages were kept within 1 m of the ground. One room in each house (veranda in House #1, carport in House #2 and the space under the house in House #3) was untreated and used as a control. In short, these designs involved ten mosquitoes per cage, two cages per room per trial, four rooms per house and three houses.

Biting activity was measured by placing a human hand on one side of the cage for 2 min and counting the maximum number of mosquitoes that probed on the hand at the same time. To prevent blood feeding and to provide a contrasting visual background against which to count mosquitoes, the hand was covered with a white sock owned by that investigator and worn the previous day without washing.

Counts of knockdown and landings were taken after 10, 30, 60 and 120 min of continuous exposure. Between trials, the emanators were removed and the rooms and cages were ventilated for ≥ 2 h with fans on and open doors and windows. The greatest number of trial repetitions possible were carried out given the available time per house; there were 5 trials carried out in the rooms of House #1, 12 in House #2 and 6 in House #3. Temperature and humidity were measured in each room, and an RS-1340 hotwire anemometer (RS Components Australia) was used to measure air speed in each room for each count by placing the probe in between the emanator and the cage.

### Cumulative mortality trials

These additional trials took place in House #2. Temperature ranged between 22 °C and 31 °C. Mosquitoes and cages were set up as above. The rooms used were Bedroom #2 (control), bedroom #3 and the lounge (Table 1). In each treatment room 2 cages were placed 1 m away from the emanator and two cages placed 3 m from the emanator. The control room had similar placement but no emanator. There were 10 mosquitoes in each cage. Biting activity and knockdown were measured pre-exposure and after 1, 2, 6, 24, 29, 48, 53 and 72 h exposure.

### Residual effects of metofluthrin exposure on cages

These trials took place in bedroom 3 in House #2 Mosquitoes and cages were set up broadly as above. An emanator was placed in the room for 1 h to ensure dissemination of the volatile molecule in the room. After that time three empty mosquito cages were exposed within that room for 2 h. After 2 h, the cages were taken outside, dismantled and thoroughly ventilated. They were then taken to an unexposed space (the area under the house), rebuilt and further evaluated for any impact on mosquito behaviour. Ten female *Ae. aegypti* were added to each exposed cage and probing and knockdown were monitored over a 2 h period. As controls, three unexposed cages were evaluated in the same way. The experiment was repeated twice.

### Data analysis

Landing rate and knockdown data were analysed using a generalized estimating equation (GEE; IBM SPSS Statistics 22) with binomial distribution and logit-link. The dependent variable was the number of events (e.g. numbers knocked down or landing) out of 10 trials. Other predictors entered into the model were time (including pre-exposure, which served as a negative control), distance from emanator (1 m or 3 m), volume of the room and all 2-way interactions between them. The working correlation matrix was assumed to be unstructured. Estimated means were calculated, and their differences were analysed for statistical significance using Fisher’s LSD to adjust for multiple comparisons.

## Results

### Efficacy trials

**Fig. 2:**
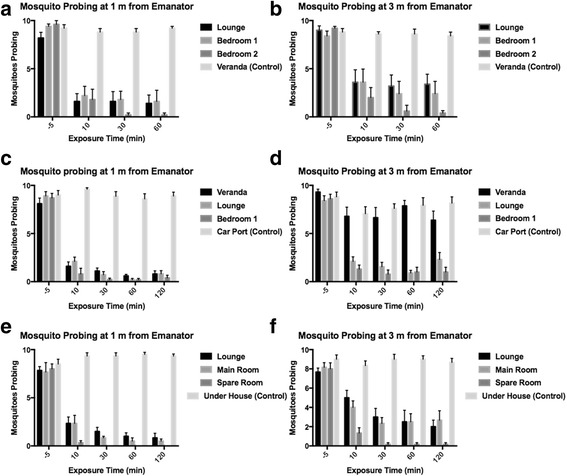
*Aedes aegypti* probing on a human hand after exposure to metofluthrin emanators. Height of bars represents the mean ± SE of probing mosquitoes (*n* = 10 in all trials). **a**-**b**, House #1. **c**-**d**, House #2. **e**-**f**, House #3. See Table 1 for dimensions and volumes of rooms

**Fig. 3:**
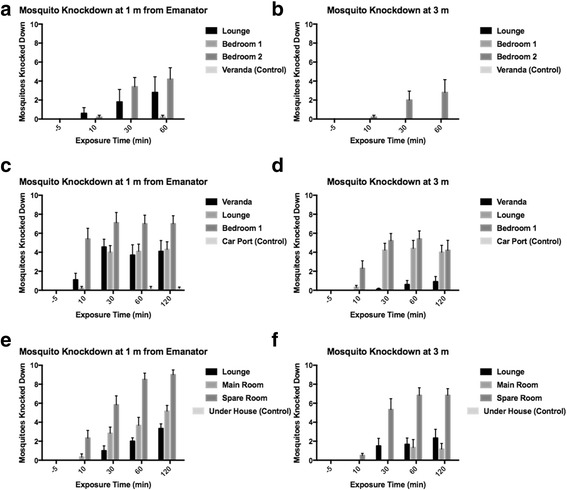
*Aedes aegypti* knockdown after exposure to metofluthrin emanators at two distances in three houses. Height of bars represents the mean ± SE of knocked down mosquitoes (*n* = 10 in all trials). Knocked down was defined as could not fly or walk after the cage was gently tapped against the floor. **a**-**b**, House #1. **c**-**d**, House #2. **e**-**f**, House #3. See Table 1 for dimensions and volumes of rooms

Overall, metofluthrin was found to reduce landing rates over time, while landing rates in unexposed controls tended to remain between 80 and 100% (Fig. 2). Landing rates were even reduced in an open area (veranda) at 1 m (Fig. 2c). Control and pre-exposure mortality was ≤ 10% in all cases (Fig. 3). The distribution of mosquitoes in exposed cages was not formally quantified, but, anecdotally, it was observed that mosquitoes were distributed around each cage approximately uniformly, i.e. did not favour one side or another. Wind speed varied between 0 and 0.91 m/s (mean ± standard error 0.05 ± 0.00) and did not correlate with landing rates or knockdown.

**Fig. 4:**
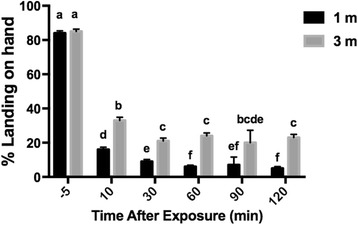
Mean landing rates of *Aedes aegypti* on a human hand with metofluthrin emanators. Estimated means (± SE) based on generalized estimating equation. Values with identical lowercase letters are not significantly different based on pairwise comparisons (using Fisher’s LSD to control for multiple comparisons)

According to the GEE, exposure time and room volume were significant predictors of landing rates (Table 2). Distance was not significant as a main effect, but its interaction with room volume was significant, i.e. metofluthrin-related inhibition in mosquito landing rates was smaller with increasing room volume, and this effect was less pronounced when the distance was 1 m (Table 2). Landing rates were always lower at 1 m than 3 m. At both 1 m and 3 m, the largest decreases in landing rate happened in the first 10 min of exposure. At 1 m, the landing rate continued to fall until 60 min. At 3 m, it fell until 30 min then stayed constant (Fig. 4). Landing rates at 1 m were reduced in the only outdoor area to be metofluthrin exposed (Fig. 2c).

**Table 2:** Estimates of parameters affecting *Aedes aegypti* landing rates in the presence of metofluthrin emanators

Parameter	Value	Standard error	95% Confidence interval	Wald *χ* ^2^	*df*	*P*-value
Intercept	-2.185	0.4663	-3.099–1.271	21.961	1	< 0.0001
Time (-5 min)	3.145	0.4707	2.222–4.067	4.067	1	< 0.0001
Time (10 min)	0.667	0.4658	-0.245–1.580	2.053	1	0.152
Time (30 min)	0.077	0.4686	-0.842–0.995	0.027	1	0.870
Time (60 min)	0.227	0.4673	-0.689–1.143	0.235	1	0.628
Time (120 min)	0.168	0.4702	-0.753–1.090	0.128	1	0.720
Distance (1 m)	-0.489	0.8762	-2.206–1.228	0.311	1	0.577
Room volume	0.015	0.0016	0.012–0.018	85.085	1	< 0.0001
Distance (1 m) * room volume	-0.014	0.0028	-0.020– -0.009	25.930	1	< 0.0001

Parameter estimates are from a generalized estimating equation. All main effects are listed. Only significant two-way interactions are shown

**Fig. 5:**
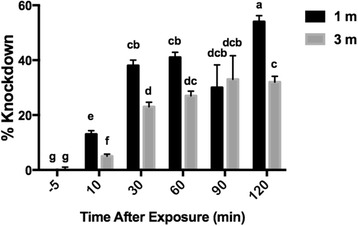
Estimated means of *Aedes aegypti* knockdown in the presence of metofluthrin emanators. Estimated means (± SE) based on generalized estimating equation. Values with identical lowercase letters are not significantly different based on pairwise comparisons (using Fisher’s LSD to control for multiple comparisons)

In the knockdown model, the significant factors were exposure time, room volume and an interaction between time and distance (Table 3). Knockdown in an outdoor veranda at 3 m was not different from untreated controls (Fig. 3d). Emanators tended to knockdown more mosquitoes at 1 m than 3 m. At both distances, most knockdown had occurred by 30 min, although there were small increases between 60 and 120 min in both cases. At 120 min, knockdown reached a maximum of 33% at 3 m and 54% at 1 m (Fig. 5).

**Table 3:** Estimates of parameters effecting *Aedes aegypti* knockdown in the presence of metofluthrin emanators

Parameter	Value	Standard error	95% Confidence interval	Wald *χ* ^2^	*df*	*P*-value
Intercept	0.057	0.3966	-0.720–0.835	0.021	1	0.885
Time (-5 min)	-5.848	1.0737	-7.953– -3.744	29.666	1	< 0.0001
Time (10 min)	-2.206	0.4251	-3.039– -1.373	26.927	1	< 0.0001
Time (30 min)	-0.520	0.4002	-1.304–0.265	0.265	1	0.194
Time (60 min)	-0.268	0.3985	-1.049–0.513	0.454	1	0.500
Time (120 min)	-0.054	0.4006	-0.839–0.731	0.018	1	0.893
Distance (1 m)	-0.155	0.5576	-1.248–0.938	0.078	1	0.781
Room volume	-0.014	0.0015	-0.017– -0.011	89.309	1	< 0.0001
Time (10 min) * distance (1 m)	1.169	0.5945	0.004–2.334	3.886	1	0.049

Parameter estimates are from a generalized estimating equation. All main effects are listed. Only significant two-way interactions are shown

### Cumulative mortality trials

**Fig. 6:**
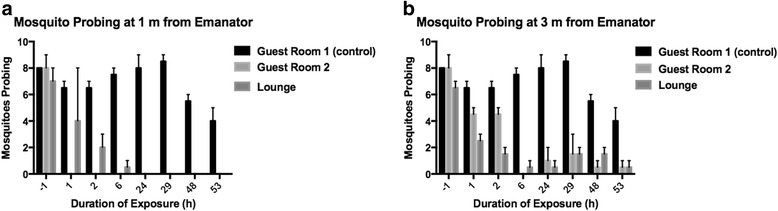
Mosquito probing in the presence of metofluthrin emanators over 48 h. Height of bars represent the mean ± SE of mosquitoes resting and probing on a human hand within a 2 min period (*n* = 10 in all trials). All trials are from House #2

When caged mosquitoes were exposed to metofluthrin over an extended period, almost all mosquitoes died by 53 h (Fig. 6). Almost 100% of unexposed caged mosquitoes survived. Of the 80 exposed mosquitoes, three (out of five) survivors still attempted to feed. For those mosquitoes 3 m from the emanator, deaths began at 6 h and did not surpass 90% mortality until 48 h. At a 1 m distance, deaths began at 1 h and was ≥ 90% at 24 h. By 6 h post-exposure, landing rates in both exposed rooms fell to < 10%. At this time, mosquitoes 1 m from the emanator had stopped landing while those 3 m from the emanator continued to land at low levels (Fig. 6).

### Persistence of metofluthrin on cages

**Fig. 7:**
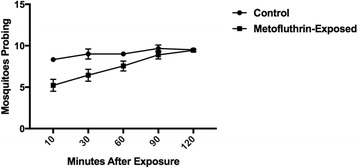
Mosquito probing in a cage that had been previously been exposed to a metofluthrin emanator for 2 h

No knockdown was observed. Probing was noticeably less frequent in cages that had previously been exposed to metofluthrin for up to an hour post-exposure, after which the proportion of mosquitoes probing became similar (Fig. 7).

## Discussion

A strength of our study is that assays were carried out in real urban spaces rather than the experimental rooms that have previously been used to demonstrate short-term reductions in *Ae. aegypti* survival and biting [16, 17]. Here we tested the efficacy of single emanators in nine different rooms in three different houses, ranging from a small bedroom (26.25 m^2^) to a large lounge (122.09 m^2^) and an outdoor veranda. This gave us the ability to assess emanator efficacy at different room volumes. Mosquitoes within 1 m of an emanator experienced the greatest inhibition in landing behaviour throughout, even in a well-ventilated outdoor veranda (Fig. 2c), suggesting that emanators may be of use in peridomestic settings (e.g. barbecue areas). The cage design of this study also allowed us a convenient way to assess feeding inhibition and knockdown over time and distance. This methodology might be of particular utility where evaluators have ethical difficulty in releasing potential disease vectors, or where released material might simply be lost to unscreened rooms. Metofluthrin emanators reduced landing rates in indoor spaces by 50–90% at 1 m and 25–90% at 3 m within 10 min of exposure irrespective of the room sizes tested here. Knockdown rates were partially dependent on room volume but ranged from 20–90% at 1 m and 0–70% at 3 m. Distance clearly had a strong effect on knockdown. This was also reflected in outdoor trials using a fan-assisted metofluthrin delivery system that only exhibited knockdown and mortality impacts at very close proximity to the device (0.3 m) [19].

The relatively low knockdown that we noted in response to exposure to these 10% w/w passive devices is relatively unimportant in terms of disease transmission given that this formulation did not appear to repel mosquitoes and therefore did not cause an increased biting risk to neighbouring, unprotected areas [20]. While the current study was not designed to evaluate repellency, other studies have demonstrated that metofluthrin-affected mosquitoes are “confused” and generally stay in the exposure area [16, 17]. Interestingly, Ponlawat et al. [21], using an outdoor, 50 m tunnel baited with a human at either end, could find no repellent or knockdown impacts of an another metofluthrin-impregnated net device (this time a 5% w/w formulation). Confused, non-biting mosquitoes are likely to starve or desiccate to death in the treated space, as emanators have been found to be effective for approximately 20 days [17]. We also showed that sustained exposure, perhaps typical of confused mosquitoes that remain in the vicinity of the emanator, will eventually lead to high mortality levels.

Generally, emanators were less effective at 3 m, both in terms of landing and knockdown. In larger (> 100 m^2^) rooms and outdoor verandas, emanator efficacy was similar to that of the control, suggesting that more than one emanator would probably be needed to improve performance in larger rooms. Although we did get some reduction in landing outdoors (in an outdoor veranda within 1 m of the emanator), the literature provides conflicting reports: the Off!® Clip-On was found to be ineffective at distances greater than 0.3 m in an outdoor test site [19] while an impregnated paper fan reduced landing rates by > 95% at a distance of 1.2 m [15]. In Cambodia, outdoor landing rates of mosquitoes (mostly *Anopheles* spp.) were reduced by 48% in the presence of a single emanator and by 67% when the collector was surrounded by 4 emanators [22]. Differences in outdoor efficacy are likely influenced by wind speed. Trials demonstrating outdoor efficacy tended to take place in wind tunnels [21], heavily wind-sheltered areas [15] or at night [22], whereas a trial showing inefficacy at distances greater than 0.3 m took place in a minimum airspeed of 5 km/h [19].

This study assayed caged mosquitoes in order to ensure sufficient baseline numbers of a mosquito species naturally present in low abundance, and in order to track knockdown over time. One drawback with this approach was the inability to observe natural free-flight behaviour [16, 17], which could answer questions such as will free-flying mosquitoes remain in the metofluthrin treated area for longer than 24 h, are free-flying mosquitoes even more likely to exhibit decreased landing (cages for mosquitoes into close proximity to a blood source - no host seeking behaviour over distance is required) or how does mosquito harbourage affect emanator effects on mosquito biting. Rapley et al. [16] found that free-flight mosquitoes stopped biting by 1 h. Here, we found a small number of mosquitoes continued to bite after 48 h, but this result should be confirmed in free-flight mosquitoes. An area of concern for dengue management is insecticide resistance. The use of residual pyrethroids is a mainstay of dengue control in Australia and is threatened by the potential evolution, incursion or establishment of pyrethroid-resistant mosquitoes. Despite some resistance reported in a similar insecticide transfluthrin [23], we have found in preliminary studies that *Ae. aegypti* carrying pyrethroid-resistant genes are still affected by metofluthrin in terms of their biting behaviour (Rigby, Devine et al., unpublished data). Dengue, Zika and other mosquito-borne mosquitoes continue to be a serious threat to public health. Upcoming technologies such as *Wolbachia* [18, 24], the sterile insect technique [25] and genetically-modified mosquitoes [26] are promising, but trials to demonstrate reduction in disease incidence have yet been carried out, and these methods are operationally not ready for widespread application. In the meantime, novel, insecticide-based interventions play a critical role in controlling disease outbreaks, and volatile pyrethroids such as metofluthrin may improve the operational efficiency of current public health intervention.

## Conclusions

Insecticide treatment of larval and adult *Aedes aegypti* is the primary method of dengue control in the absence of effective drugs or vaccines, but a considerable challenge of this method is the slow application time. We found that emanators, which passively dispense the volatile pyrethroid metofluthrin, placed in residential rooms decrease biting rates of *Ae. aegypti* by as much as 90% when placed within 1 m. We recommend a second emanator for larger rooms. Metofluthrin emanators did not kill many mosquitoes - approximately 50% within 1 m - but this would not undermine the efficacy of metofluthrin, as mosquitoes that are not biting will not contribute to dengue (or other arbovirus) transmission. Metofluthrin emanators should not replace traditional indoor residual spraying, but our results demonstrate that it could be a useful supplementary tool.

## Abbreviations

GEE: Generalized estimating equation
LSD: Least significant difference
SE: Standard error
